# Storage of firearms in vehicles: findings from a sample of firearm owners in nine U.S. states

**DOI:** 10.1186/s40621-024-00525-1

**Published:** 2024-09-10

**Authors:** Alexander Testa, Daniel C. Semenza, Michael Anestis

**Affiliations:** 1 Department of Management, Policy and Community Health, University of Texas Health Science Center at Houston, 1200 Pressler Street, Houston, TX 77030 USA; 2 Department of Sociology, Anthropology, and Criminal Justice, Rutgers University, Camden, NJ USA; 3 Department of Urban-Global Public Health, School of Public Health, Rutgers University, Piscataway, NJ USA; 4 New Jersey Gun Violence Research Center, Rutgers University, Piscataway, NJ USA

**Keywords:** Firearms, Firearm storage, Vehicles, Firearm theft, Firearm purchasing

## Abstract

**Background:**

In recent years, there has been a growing number of thefts of firearms stored in vehicles. Despite this trend, there is limited research on firearm storage patterns in vehicles in the United States. This study investigates these storage patterns and evaluates the relationship between the surge in firearm purchases after March 2020 and the practice of storing firearms in vehicles.

**Methods:**

Firearm storage practices were classified into four categories: (a) no vehicle storage, (b) locked vehicle storage only, (c) unlocked vehicle storage only, and (d) both locked and unlocked vehicle storage. Multinomial logistic regression analyses were conducted to determine the association between vehicle firearm storage practices and the main independent variable (firearm purchases since March 2020), adjusting for covariates.

**Results:**

Those who purchased a firearm since March 2020 were significantly more likely to store at least one firearm in a vehicle unlocked only (RRR = 2.41, 95% CI 1.45–3.99) or both locked and unlocked (RRR = 2.57, 95% CI .180–3.67) compared to the reference category of no vehicle storage.

**Conclusion:**

Individuals who purchased a firearm after March 2020 were more likely to report storing a firearm in a vehicle. Given the limited research on patterns of firearm storage in vehicles, these findings provide novel evidence suggesting that firearm purchases following the March 2020 firearm purchasing surge may have fomented behaviors that increased the likelihood of firearm storage in automobiles. Moving forward, there is a need for additional quantitative and qualitative research that can better understand patterns and motivations of firearm storage in vehicles.

## Introduction

The United States (U.S.) is distinguished by its high level of firearm ownership, with an estimated 378 million firearms in circulation as of 2022 (Mascia 2023). Starting around March 2020, a substantial increase in firearm sales began, with millions of new firearms entering circulation during the initial months of the COVID-19 pandemic alone (Schleimer et al. 2021; Crifasi et al. 2021), including many purchased by first-time owners (Miller et al. 2022). This surge in firearm ownership has raised questions about recent firearm purchases and firearm storage practices, with some research suggesting that nearly 40% of new firearm owners who acquired firearms in response to the pandemic reported storing at least one firearm unlocked (Lyons et al. 2021). An emerging yet insufficiently explored aspect of firearm storage involves the storage of firearms in vehicles. (Tucker et al. 2023)

Although there remains very little research on the motivations for storing a firearm in a vehicle, individuals may choose to store firearms in vehicles for various reasons, ranging from personal security concerns to accessibility while traveling (Freskos 2016). The perception of a vehicle as an extension of one’s personal space can also influence the decision to store firearms within vehicles. Additionally, legal restrictions on firearm carrying and the lack of secure storage options at destinations may compel firearm owners to leave firearms in vehicles. However, firearms stored in vehicles are particularly susceptible to theft (Freskos 2016; Hemenway et al. 2017), subsequently increasing the likelihood of these weapons being used for criminal activity or circulated on illegal firearm markets (Fausset 2023; Cook 2018; Szkola et al. 2024). Indeed, estimates suggest that approximately 380,000 firearms are stolen annually (Hemenway et al. 2017), and vehicle thefts account for an increasing share of thefts, tripling between 2013 and 2023 (Szkola et al. 2024). A recent report from Everytown For Gun Safety finds that firearm thefts from vehicles represent the largest source of stolen firearms, with an at least one gun stolen from a vehicle every nine minutes in the United States on average (Szkola et al. 2024). In response, The White House issued guidance and advocated for legislation mandating secure firearm storage in vehicles in 2024 (The White House 2024). Additionally, at the state level, recent legislative action has addressed firearms stolen from vehicles, such as legislation in Colorado that requires secure firearm storage. (Fields et al.)

Despite its significance, research on vehicle firearm storage remains sparse. A study among 408 adult male firearm owners revealed that over 40% reported storing firearms in their vehicles on at least an occasional basis (Tucker et al. 2023). A nationally representative survey on storage practices of U.S. firearm owners in 2016 found that 11% of firearm owners reported storing all or some of their firearms in a vehicle when at home (Crifasi et al. 2018). Data from a survey conducted in the summer of 2022 across five states involving 2,152 adults found that 7.1% of participants reported locked storage of firearms in vehicles (Anestis et al. 2023). In a national study of Black and American Indian and Alaska Native (AIAN) adults, researchers found that 10% of Black firearm owners and 13% of AIAN firearm owners say they “almost always” or “always” store at least one firearm in an unlocked vehicle (Anestis et al. 2024).

Using data from a sample of firearm owners in nine U.S. states, the current study examines the patterns of firearm storage in vehicles, focusing on the impact of firearm purchases following the March 2020 firearm purchasing surge on the likelihood of vehicle storage.

## Methods

### Data

We collected state-representative data from non-institutionalized adults in nine states (M.S., NJ, CO, TX, MN, WA, PA, OH, and F.L.) in collaboration with Ipsos KnowledgePanel, the largest probability-based online panel of adults in the U.S. Data were collected in June and July 2023. Respondents were invited by email to complete surveys, and a follow-up invitation was sent three days after the initial invitation for non-responders. The median survey completion time was 25 min. A total of 13,568 surveys were fielded, and 8,490 were completed (completion rate = 63%). The final sample of qualified and completed surveys was 7,785 (qualified rate = 92%). The survey instrument and study were approved by the Rutgers University Institutional Review Board. Design weights were created using an iterative proportional fitting procedure which raked weights to geodemographic distributions of state populations based on the following 2021 American Community Survey benchmarks: race and ethnicity, gender, education, and household income. These weights were then trimmed to remove outliers at the extreme upper and lower tails of the weight distribution and scaled to add up to the total sample size of all qualified respondents. This final trim and scale step ensures no unusually high or low weights disproportionately bias the survey results because of non-response adjustment or post-stratification while ensuring representativeness.

### Measures

*Firearm access.* Respondents were asked, “Is there typically a firearm stored in or around your home?” All respondents who indicated “yes” were included in our sample of firearm owners for this study.

*Firearm storage in vehicle.* Firearm owners were asked to indicate how often they utilize specific firearm storage practices, ranging from “never (0%)” to “always (100%).” We specifically measured locked vehicle storage using the item, “Store at least one firearm in a vehicle, in a locked container (e.g., locked glove box),” and unlocked vehicle storage using the item, “Store at least one firearm in a vehicle, not in a locked container.” Since respondents could choose multiple storage methods, we created a categorical variable using the following categories to ensure we accounted for all overlap: no vehicle storage (0), locked vehicle storage only (1), unlocked vehicle storage only (2), and both locked and unlocked vehicle storage (3).

*Purchased firearm since March 2020*. We measured whether respondents had purchased a firearm since March 2020 by asking, “Have you purchased a firearm since March 2020?”.

*Covariates.* All models account for a range of demographic control measures, including sex, age group, education level, race and ethnicity, household income, marital status, employment status, military status, home rental vs. ownership, metropolitan area status. A variable is also included for perceived safety measured using agreement with nine items (alpha = 0.942) ranging from strongly agree (1) to strongly disagree (7) [range = 0–63]. Items included statements like, “I feel terrified that I may someday be the victim of a robbery,” “I am afraid of being physically assaulted,” and “I do not feel safe being home alone.” Greater scores indicated higher perceived safety. Finally, we controlled for state of residence to account for state-level policies or firearm ownership differences that might impact the results (Schell et al. 2020). For instance, New Jersey and Washington are the only of these nine states at the time of data collection that had a law requiring the secure storage of handguns in unattended vehicles (see N.J. Stat. Ann. § 2C:58–4.6(b)(2) and Wash. Rev. Code Ann. § 9.41.050). Weighted descriptive statistics for all measures are included in Table 1.

**Table 1:** Weighted descriptive statistics—firearm Owners (N = 3,063)

	N	%		N	%
Firearm stored in vehicle			Marital status		
No vehicle storage	2196	73	Married	1947	64
Locked vehicle storage only	158	5	Widowed	124	4
Unlocked vehicle storage only	279	9	Divorced/separated	326	11
Both locked and unlocked vehicle storage	387	13	Never married	623	21
Purchased firearm since March 2020	707	23	Employment status		
Female	1479	49	Full time	1487	49
Age			Part time	436	14
18–29	382	13	Not working	1098	36
30–44	790	26	Current/former military	464	15
45–59	834	28	Rent home	402	13
60 +	1015	34	Live in metropolitan area	2542	84
Education			State		
No HS	158	5	TX	908	30
H.S. degree	906	30	NJ	92	3
Some college	989	33	PA	403	13
Bachelors or more	968	32	OH	346	11
Race and ethnicity			MN	192	6
Non-Hispanic White	2135	71	FL	555	18
Non-Hispanic Black	236	8	MS	126	4
Hispanic	457	15	CO	172	6
Non-Hispanic raOther/2 +	192	6	WA	226	7
Household income				M	SD
< $24,999	204	7	Perceived safety	50.34	0.31
$25,000 to $74,999	983	33			
$75,000 to $149,999	1190	39			
$150,000 +	645	21			

### Analytic strategy

Our analytic sample consisted of firearm owners, comprising about 40% of our total sample (N = 3,119). We used listwise deletion to account for missing data among a small number of respondents, leading to a final sample of 3,063 firearm owners (1.7% missing data). We used multinomial logistic regression models to regress whether individuals store their firearms locked in a vehicle, unlocked in a vehicle, or both unlocked and locked in a vehicle, compared to no vehicle storage on the independent variable (firearm purchase since March 2020), controlling for covariates. All analyses were conducted in Stata 18.

## Results

As shown in the weighted descriptive statistics depicted in Table 1, about 73% of the sample reported not storing a firearm in their vehicle, whereas 5% reported storing a firearm locked only in a vehicle, 9% reported storing a firearm unlocked only in a vehicle, and 13% reported storing a firearm both locked and unlocked in a vehicle. Roughly a quarter of firearm owners reported they purchased a firearm since March 2020. Additionally, descriptive statistics and cross-tabulations of firearm surge purchasing and vehicle firearm storage by demographic characteristics are included in the Appendix.

Table 2 depicts the results of the multinomial logistic regression models. Those who purchased a firearm since March 2020 were significantly more likely to have stored at least one firearm in a vehicle unlocked only (RRR = 2.41, 95% CI 1.45–3.99) or unlocked and locked (RRR = 2.57, 95% CI 1.80–3.67) compared to the reference category of no vehicle storage.

**Table 2:** Multinomial logistic regression of the association between purchased a firearm since 2020 and vehicle fiream storage [reference category = no vehicle storage] (N = 3,063)

	OR	SE	CI	
	Unlocked only			
Purchased firearm since March 2020 (ref = no)	2.41***	0.62	1.45	3.99
	Locked only			
Purchased firearm since March 2020 (ref = no)	1.49	0.35	0.94	2.37
	Unlocked and locked			
Purchased firearm since March 2020 (ref = no)	2.57***	0.47	1.80	3.67

p < .001 ***

Regression analysis adjusts for sex, age group, education level, race and ethnicity, household income, marital status, employment status, military status, home rental vs. ownership, metropolitan area status, perceived safety, and state of residence

In supplemental analyses (see Appendix A), we used a more stringent coding of storage in a vehicle to only include those that indicate “almost always” or “always” storing a firearm locked or unlocked in a vehicle. The results were very similar to those presented in the main analysis, although these models displayed statistically significant associations for unlocked only (RRR = 2.88, 95% CI 1.62–5.10), locked only (RRR = 2.02, 95% CI 1.16–3.53) and both unlocked and locked (RRR = 2.18, 95% CI 1.09–4.39) compared to no vehicle storage. We further assessed whether first-time firearm purchasing during the March 2020 firearm purchasing surge (“Was the firearm(s) you purchased since March 2020 the first firearm(s) you have ever acquired?”) and more recent firearm purchasing (“Have you purchased a firearm since March 2022?”) were associated with vehicle firearm storage. In the subsample of those who purchased a firearm since March 2020, we found that neither first-time purchases during the March 2020 firearm purchasing surge nor more recent firearm purchases since March 2022 were statistically associated with having stored a firearm locked or unlocked in a vehicle (see Appendices B and C).

## Discussion

Using a sample of firearm owners from nine states, the findings of our study shed new light on firearm storage patterns by demonstrating that individuals who purchased a new firearm since March 2020 were significantly more likely to report storing a firearm in their vehicles either unlocked or both unlocked and locked relative to not storing firearms in their vehicle. These findings correspond with prior research showing that new firearm owners are significantly more likely to engage in unsecure storage practices (Lyons et al. 2021) and also correspond with trends of an uptick in firearm thefts from vehicles in the United States in recent years. (Hemenway et al. 2017; Fausset 2023; Szkola et al. 2024)

The core finding that purchasing a firearm since March 2020 was associated with storing a firearm in a vehicle—either locked or unlocked—can potentially be understood within the context of the social and psychological stresses precipitated by the COVID-19 pandemic (Salari et al. 2020). The onset of the pandemic in March 2020 heralded rapid and unprecedented perceptions of uncertainty, fear, and threats to personal safety, partly due to societal disruptions, economic instability, and increased changes to patterns of crime (Galea and Abdalla 2020; Meyer et al. 2022; Coelho et al. 2020). These conditions may have heightened security concerns among those purchasing a firearm during this period, who desired self-protection when traveling, thereby leading to increased use of firearm storage in a vehicle, which fomented storage patterns that become ingrained over time.

Even so, given the limited data on storing vehicles in firearms, it is challenging to understand the reasons behind this behavior fully. Addressing this issue necessitates further research to understand the motivations for storing firearms in vehicles and efforts that can inform the practices. In particular, there is a pressing need for further qualitative and quantitative research to investigate the motivations and decision-making processes behind this practice to understand the nuances of why firearm owners opt to store their firearms in vehicles.

The findings point to actions that can be taken surrounding the practice of firearm storage in vehicles to reduce the risk of harm stemming from the practice, such as firearm theft (Hemenway et al. 2017). Public awareness campaigns can be a critical tool in educating firearm owners about the dangers of storing unsecured firearms in vehicles, emphasizing the community and personal safety benefits of proper storage practices. Even so, while campaigns focusing on reducing firearm storage in vehicles have appeared in several jurisdictions in recent years (DelPonte and New 2014; Diamante 2020; Zavala 2023), research is needed to elucidate the best messaging and outreach practices and whether such efforts correspond with a change in storage practices. The firearm and automobile industries can also play a vital role in this context by developing and promoting secure storage solutions tailored for vehicle use and encouraging adoption through incentives that break down cost barriers. Given research demonstrating a sizeable demand for biometric in-vehicle locking devices among firearm owners nationwide (Betz et al. 2023), such initiatives can potentially leverage a large market to promote more secure storage practices.

### Limitations

There are limitations in the current study worth noting. First, the findings are from firearm owners in nine states and are not generalizable beyond these states. Although there was a 63% response rate for the survey, weighting was still used to ensure the representativeness of the sample across the nine states that were included. Although the states are not representative of the full U.S., they are diverse regarding region, political composition, firearm ownership, and firearm legislation. Second, because the data are from a cross-sectional survey, the results are described as associational due to the possibility of unmeasured confounding. Third, we do not know the precise timing of when a firearm was purchased or the exact motivations for buying a recent firearm, which may impact the decision to store a firearm in a vehicle. Fourth, there are limitations regarding the details of vehicle firearm storage that can be explored in greater depth in future research. For instance, locking a firearm in a container such as a glove box would be more challenging to remove from a vehicle than locking a firearm in a locked box visible in the back seat. Accordingly, future research that examines the granularity of in-vehicle storage methods and the correlates of such methods with greater granularity would be highly valuable. Fifth, cell sizes prohibited a detailed examination of how the results varied by demographic characteristics. However, investigating heterogeneity in patterns of vehicle firearm storage is an important direction for future research. Finally, responses to firearm storage patterns may be subject to recall or social desirability bias.

## Conclusion

The findings demonstrate that individuals who purchased a firearm since the March 2020 firearm purchasing surge were significantly more likely to report storing a firearm in a vehicle either unlocked or both locked and unlocked. These findings highlight crucial areas for further investigation, emphasizing the need for research investigating the intricate patterns and underlying motivations behind storing firearms in vehicles. Building knowledge in this area is instrumental in developing effective strategies to mitigate risks associated with firearm storage in vehicles.

## Data Availability

The datasets generated during and/or analyzed during the current study are available from the corresponding author upon reasonable request.

## Appendix

### Appendix A: Multinomial logistic regression of the association between purchased a firearm since 2020 and vehicle fiream storage (almost always/always) [reference category = no vehicle storage] (N = 3,063)

**Table:**

	OR	SE	CI	
	Unlocked only			
Purchased firearm since March 2020 (ref = no)	2.88***	0.84	1.62	5.10
	Locked only			
Purchased firearm since March 2020 (ref = no)	2.02*	0.57	1.16	3.53
	Unlocked and locked			
Purchased firearm since March 2020 (ref = no)	2.18*	0.78	1.09	4.39

p < 0.001 ***; p < 0.05 *

Regression analysis adjusts for sex, age group, education level, race and ethnicity, household income, marital status, employment status, military status, home rental vs. ownership, metropolitan area status, perceived safety, and state of residence

### Appendix B: Multinomial logistic regression of the association between first time purchase during March 2020 surge and vehicle fiream [reference category = no vehicle storage] (N = 616)

**Table:**

	OR	SE	CI	
	Unlocked only			
First firearm purchased March 2020 (ref = no)	0.88	0.59	0.24	3.30
	Locked only			
First firearm purchased March 2020 (ref = no)	1.55	0.75	0.60	4.00
	Unlocked and locked			
First firearm purchased March 2020 (ref = no)	1.17	0.82	0.30	4.62

Regression analysis adjusts for sex, age group, education level, race and ethnicity, household income, marital status, employment status, military status, home rental vs. ownership, metropolitan area status, perceived safety, and state of residence

### Appendix C: Multinomial logistic regression of the association between purchased a firearm since March 2022 and vehicle firearm [reference category = no vehicle storage] (N = 616)

**Table:**

	OR	SE	CI	
	Unlocked only			
Firearm purchased since March 2022 (ref = no)	0.96	0.52	0.34	2.76
	Locked only			
Firearm purchased since March 2022 (ref = no)	0.87	0.35	0.40	1.91
	Unlocked and locked			
Firearm purchased since March 2022 (ref = no)	1.89	1.00	0.67	5.36

Regression analysis adjusts for sex, age group, education level, race and ethnicity, household income, marital status, employment status, military status, home rental vs. ownership, metropolitan area status, perceived safety, and state of residence

### Appendix D: Vehicle Firearm Storage by Racial Group (N = 3,155)

**Table:**

	Total	White	Black	Hispanic	Other/2 +
No vehicle storage	2264 (0.73)	1682 (0.76)	142 (0.59)	301 (0.63)	140 (0.69)
Unlocked only	160 (0.05)	111 (0.05)	14 (0.06)	30 (0.06)	6 (0.03)
Locked only	292 (0.09)	170 (0.08)	33 (0.14)	75 (0.16)	14 (0.07)
Both	403 (0.13)	239 (0.11)	53 (0.22)	69 (0.15)	42 (0.21)
Count (%)					

Survey- adjusted Pearson's Chi2 statistically significant for all groups (p =  < 0.000)

### Appendix E: Vehicle firearm storage by sex group (N = 3,155)

**Table:**

	Total	Male	Female
No vehicle storage	2264 (0.73)	1139 (0.71)	1125 (0.74)
Unlocked only	160 (0.05)	107 (0.07)	53 (0.04)
Locked only	292 (0.09)	156 (0.10)	136 (0.09)
Both	403 (0.13)	197 (0.12)	206 (0.14)
Count (%)			

Survey- adjusted Pearson's Chi2 statistically significant for all groups (p = 0.0497)

### Appendix F: Vehicle Firearm Storage by Age Group (N = 3,155)

**Table:**

	Total	18–29	30–44	45–59	60 +
No vehicle storage	2264 (0.73)	278 (0.68)	555 (0.69)	626 (0.73)	805 (0.77)
Unlocked only	160 (0.05)	38 (0.09)	22 (0.03)	48 (0.06)	51 (0.05)
Locked only	292 (0.09)	41 (0.10)	93 (0.12)	73 (0.09)	85 (0.08)
Both	403 (0.13)	50 (0.12)	138 (0.17)	113 (0.13)	101 (0.10)
Count (%)					

Survey-adjusted Pearson's Chi2 statistically significant for all groups (p = 0.0138)

### Appendix G: Surge purchasers since March 2020 by racial group (N = 3,135)

**Table:**

	Total	White	Black	Hispanic	Other/2 +
No	2371 (0.77)	1718 (0.79)	162 (0.67)	350 (0.74)	141 (0.73)
Yes	721 (0.23)	466 (0.21)	79 (0.33)	124 (0.26)	52 (0.27)

Survey-adjusted Pearson's Chi2 not statistically significant (p = 0.0721)

### Appendix H: Surge purchasers since March 2020 by sex group (N = 3,135)

**Table:**

	Total	Male	Female
No	2371 (0.77)	1083 (0.68)	1288 (0.85)
Yes	721 (0.23)	503 (0.32)	219 (0.15)

Survey-adjusted Pearson's Chi2 statistically significant for all groups (p =  < 0.000)

### Appendix I: Surge purchasers since March 2020 by age group (N = 3,135)

**Table:**

	Total	18–29	30–44	45–59	60 +
No	2371 (0.77)	271 (0.68)	578 (0.72)	645 (0.76)	876 (0.84)
Yes	721 (0.23)	130 (0.32)	221 (0.28)	208 (0.24)	163 (0.16)

Survey-adjusted Pearson's Chi2 statistically significant for all groups (p =  < 0.000)
